# Efficient Ternary Organic Photovoltaic Films for Fast Exciton Separation to Generate Free Radicals for Wastewater Treatment

**DOI:** 10.1002/EXP.70001

**Published:** 2025-02-04

**Authors:** Linji Yang, Ciyuan Huang, Yang Zhou, Libin Zhang, Ke Sun, Houjin Luo, Yinna Liang, Yilin Wang, Tao Yang, Wei Ma, Donglou Ren, Cong Liu, Heng Zhang, Kai Chen, Hongxiang Zhu, Jianhua Xiong, Bingsuo Zou, Shuangfei Wang, Tao Liu

**Affiliations:** ^1^ School of Chemistry and Chemical Engineering Guangxi Key Laboratory of Processing for Non‐ferrous Metals and Featured Materials State Key Laboratory of Featured Metal Materials and Life‐cycle Safety for Composite Structures School of Resources Environment and Materials Guangxi University Nanning China; ^2^ Department of Biochemistry and Cell Biology Youjiang Medical University for Nationalities Baise China; ^3^ State Key Laboratory for Mechanical Behavior of Materials Xi'an Jiaotong University Xi'an China; ^4^ Centre for Mechanical Technology and Automation Department of Mechanical Engineering University of Aveiro Aveiro Portugal

**Keywords:** insertion strategy, morphology, organic solar cells, power conversion efficiency, water purification

## Abstract

Given the effectiveness of organic pollutants photodegradation and the excellent photovoltaic nature of organic solar cells (OSCs), this work first innovatively integrated the cross‐fields of OSCs and environmental photocatalysis. Using knowledge of OSC morphology, an insertion strategy involved adding a suitable quantity of guest acceptor (Y6‐O) to the PM6 donor polymer and BTP‐2F‐ThCl host small molecule acceptor system. Y6‐O leads to tighter π–π packing, reduced domain size, and improved domain purity, resulting in favorable morphology for charge generation and transfer in devices and an improved power conversion efficiency (PCE) from 17.1% to 18.1%. Moreover, terpolymer organic photovoltaic films were applied to wastewater treatment, gaining ions Sb(III) and Sb(V) removals of 100% in 15 min, and guaiacol photodegradations of 90% in 1 h. This work significantly prompts the development of organic photovoltaics and wastewater treatment and opens views for multifunctional organic photovoltaic material applications.

## Graphical abstract

For the first time, application results are reported at the intersection of organic photovoltaics and photocatalysis: An insertion strategy based on morphology modulation is proposed to reduce the π–π stacking distance, improve charge transport, and increase device efficiency. Ternary organic photovoltaic films were applied to wastewater treatment, and the removal rate of ionic Sb(III) and Sb(V) reached 100%.

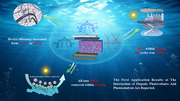

## 1 Introduction

Driven by advancement in material innovation and device engineering, the power conversion efficiency (PCE) of organic solar cells (OSCs) has exceeded 19% in many cases [1, 2, 3, 4, 5, 6, 7, 8, 9, 10, 11, 12, 13, 14, 15, 16, 17, 18, 19, 20, 21, 22, 23, 24, 25, 26, 27]. However, morphologic adjustment strategies, particularly those involving hybrids of two Y‐series small molecule acceptors (SMAs), have seldom been demonstrated thus far. The morphology of the OSC active layer, including crystallinity and phase separation behavior, plays a dominant role in determining PV performance. A high order tight π–π packing is favorable for charge transport, whereas eminent domain purity enhances efficient transport and collection [28, 29, 30, 31, 32, 33, 34, 35]. Therefore, combining different packaging properties of two structurally similar acceptors (usually exhibiting good miscibility) through a ternary strategy can result in a merging phase and appropriate co‐crystallization, leading to the desired morphology that greatly improves device efficiency and stability.

Meanwhile, although organic solar cells are an emerging technology, they still have some drawbacks that need to be overcome, including low energy conversion efficiency, easy decomposition and degradation of organic molecules, low battery performance, and high manufacturing costs. In particular, the stability of organic solar cells is deficient, and they are susceptible to moisture, oxidation, ultraviolet radiation, and heat, which can result in reduced device efficiency or failure. These shortcomings severely limit the industrialization process and the application field of organic photovoltaics. Previously, the focus has been on improving the efficiency of ternary organic photovoltaic devices, while the application of ternary organic photovoltaic film in wastewater treatment has been neglected. Compared with perovskite solar cells, which are easily degraded in water and may cause secondary pollution. The organic photovoltaic film is a polymer with a certain degree of hydrophobicity, which does not easily dissolve in water, and the preparation process is simple and relatively easy to be loaded onto the base material, which can be dedicated to the preparation of a green recyclable and pollution‐free photocatalyst [36, 37]. Currently, advanced oxidation processes (AOPs), such as the Fenton method, photocatalysis, electrochemical methods, and various combination technologies, are utilized to achieve rapid, stable, and efficient removal of refractory organic pollutants and heavy metal ions [38, 39, 40, 41, 42, 43, 44, 45, 46, 47, 48, 49, 50, 51, 52, 53]. However, most non‐organic photocatalysts exhibit some shortcomings, including low catalytic efficiency, lack of selectivity, high cost, low stability, short service life, and difficulty in adjusting energy levels. While photocatalysis is an ideal technology for wastewater treatment, it is only driven by appropriate photon energy [54, 55, 56, 57, 58, 59].

The photocatalytic processes driven solely by appropriate photon energy are an ideal technology for wastewater treatment due to their renewable energy utilization and low environmental costs [60, 61]. With the active layer materials of excellent photovoltaic performance that enables good solar energy collection, terpolymer photovoltaic thin films possess advantages such as low cost, straightforward fabrication, tunable energy level, and environmental friendliness [62, 63, 64, 65, 66]. As a new type of photocatalyst, OSCs can address the shortcomings of traditional photocatalysts. The energy level and environmental protection of terpolymer organic photovoltaic films as photocatalysts are their biggest advantages. The chemical structure of photovoltaic materials can be adjusted, which is impossible for traditional inorganic photocatalysts, to change their photoelectric performance. The flexible energy level structure can broaden the absorption spectrum, maximize light utilization, and improve the efficiency of photon capture and photocatalysis. Additionally, the preparation process does not require expensive rare metals, makes full use of biomass resources, and has a minimal impact on the environment [67, 68, 69, 70, 71, 72]. The photocatalyst can efficiently remove lead, cadmium, antimony, and copper simultaneously, and can be reused, which addresses the issues of photocatalyst loading and recycling. This provides a green, environmentally friendly, and pollution‐free treatment method for the removal of heavy metal ions and pollutants from the environment.

In this work, we constructed a ternary matrix using two Y‐series small‐molecule acceptors, BTP‐2F‐ThCl and Y6‐O, [73, 74] previously reported by others and the well‐known polymer donor PM6. Optically, Y6‐O and BTP‐2F‐ThCl exhibit complementary absorption curves, which may enhance photon collection after mixing. However, Y6‐O and PM6 show low miscibility, leading to independent flake packaging of PM6 in both horizontal and vertical directions. Despite this, the fabricated device achieved an impressive power conversion efficiency (PCE) of 18.1%, which surpasses the 17.1% PCE of the BTP‐2F‐ThCl‐based binary system, thanks to the improvements in open‐circuit voltage (*V*_OC_), short‐circuit current density (*J*_SC_), and fill factor (*FF*). Furthermore, this work represents the first and innovative application of terpolymer organic photovoltaic films in the field of wastewater treatment, demonstrating exciting removal effects. Compared with traditional photocatalysts, the novel photocatalyst prepared in this work not only has low cost, recyclability, and adjustable energy band structure but also improves photon utilization rate and achieves high removal efficiency. Moreover, it successfully overcomes the challenge that organic photoelectric materials are susceptible to moisture and oxygen. This breakthrough opens a new application direction for organic photovoltaic materials and provides a promising solution for removing heavy metal ions and organic pollutants from the environment.

## 2 Experimental

### 2.1 Materials

All materials used in this study were commercially available.

### 2.2 Preparation of Photocatalyst

PM6:BTP‐2F‐ThCl:Y6‐O (1:1.2:0, 1:1.05:0.15 and 1:0:1.2 weight ratio) was loaded on the bagasse cellulose particles. Once the membrane preparation is completed, keep dry and in dark storage.

### 2.3 Analysis and Calculation

Determination of ions antimony, cadmium, lead and copper in solution by inductively coupled plasma (ICP) spectrometry. Determination of guaiacol concentration in solution by ultraviolet–visible (UV‐Vis) spectrophotometry.

Equation (1) can be used to calculate the removal efficiency (*η*) of guaiacol, *C* is the concentration of the sample and *C*_0_ is the initial concentration.

(1) $$\eta =(\;1-C/C_{0}\;)\;/\;100\%$$

Free radical species trapping and electron spin resonance (ESR) experiments: p‐benzoquinone, isopropanol, sodium oxalate and KBrO_3_ were used as trapping agents for hydroxyl superoxide radicals (O_2_^−^), radicals (·OH), holes (h^+^) and electrons (e^−^). The above reagent concentrations were all 20 mmol L^−1^.

## 3 Results and Discussion

**FIGURE 1:**
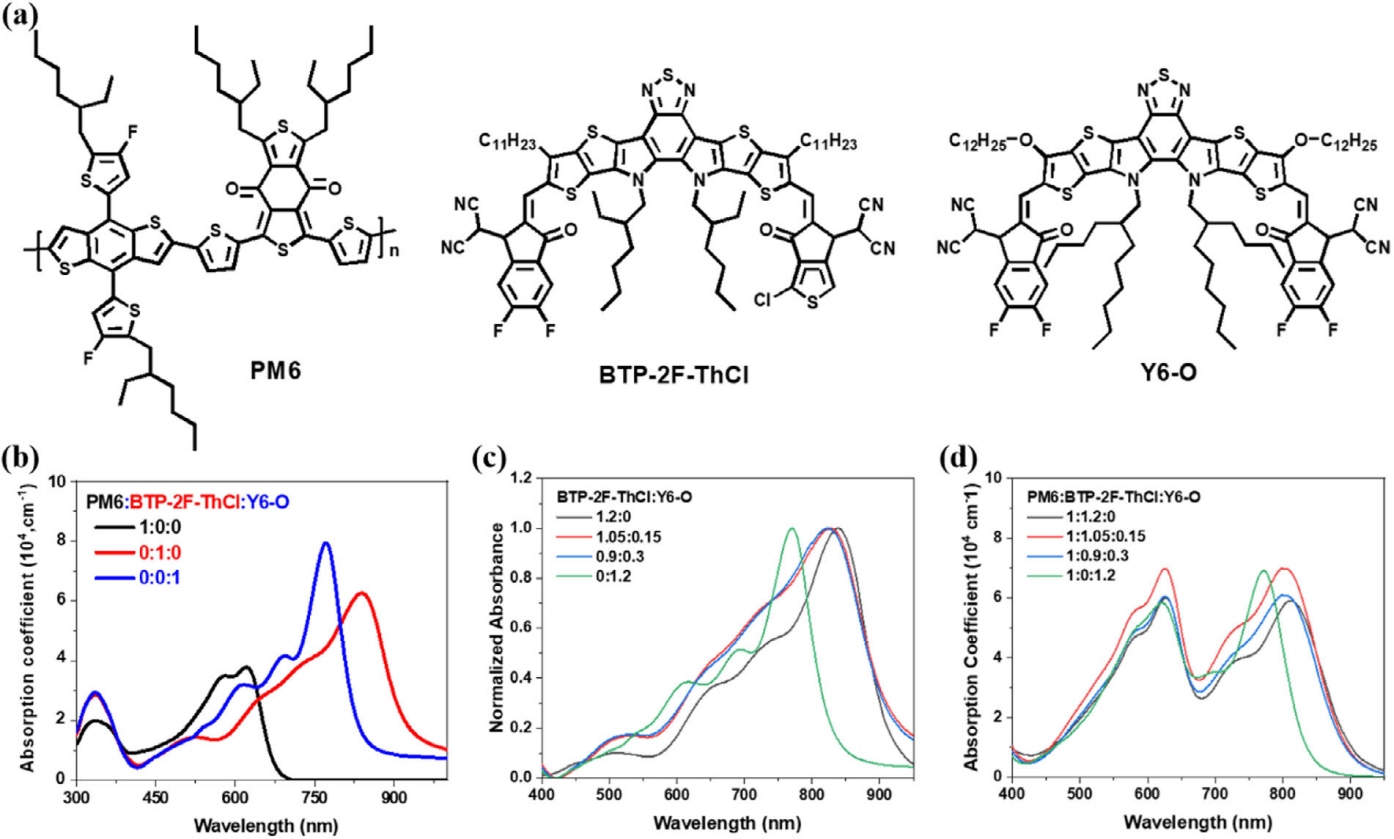
(a) Chemical structures of PM6, BTP‐2F‐ThCl and Y6‐O. UV‐vis absorption spectra of (b) neat films, (c) acceptor blending films and (d) binary and ternary films (of absorption coefficients).

The materials in this work are illustrated in Figure 1a: Their availability is identical to our previous works [5, 75]. Then our attention turns to the film's optical properties, which is studied by performing ultraviolet–visible (UV‐vis) absorption measurements. Figure 1b–d contains all testing results for different types of film combinations. Obviously, Y6‐O demonstrates a complementary absorption region for PM6 and BTP‐2F‐ThCl, so its incorporation could lead to better photon capturing (Figure 1c). This claim is further confirmed by the acceptor‐only film's absorbance comparison, where BTP‐2F‐ThCl:Y6‐O films no matter 1.05:0.15 or 0.9:0.3 exhibit broader absorption. Moreover, the donor–acceptor blend films in Figure 1d provides some interesting information: PM6:Y6‐O film has a higher acceptor peak compared with the donor peak, different from those films dominated by BTP‐2F‐ThCl. This indicates poorer miscibility between Y6‐O and PM6, which is responsible for Y6‐O's stronger independent aggregation and crystallization, which are reflected by the absorption characteristics. Therefore, Y6‐O and BTP‐2F‐ThCl contain different morphological features in films when blending with PM6, bearing the possibility of achieving tuned morphology once combined with each other.

**FIGURE 2:**
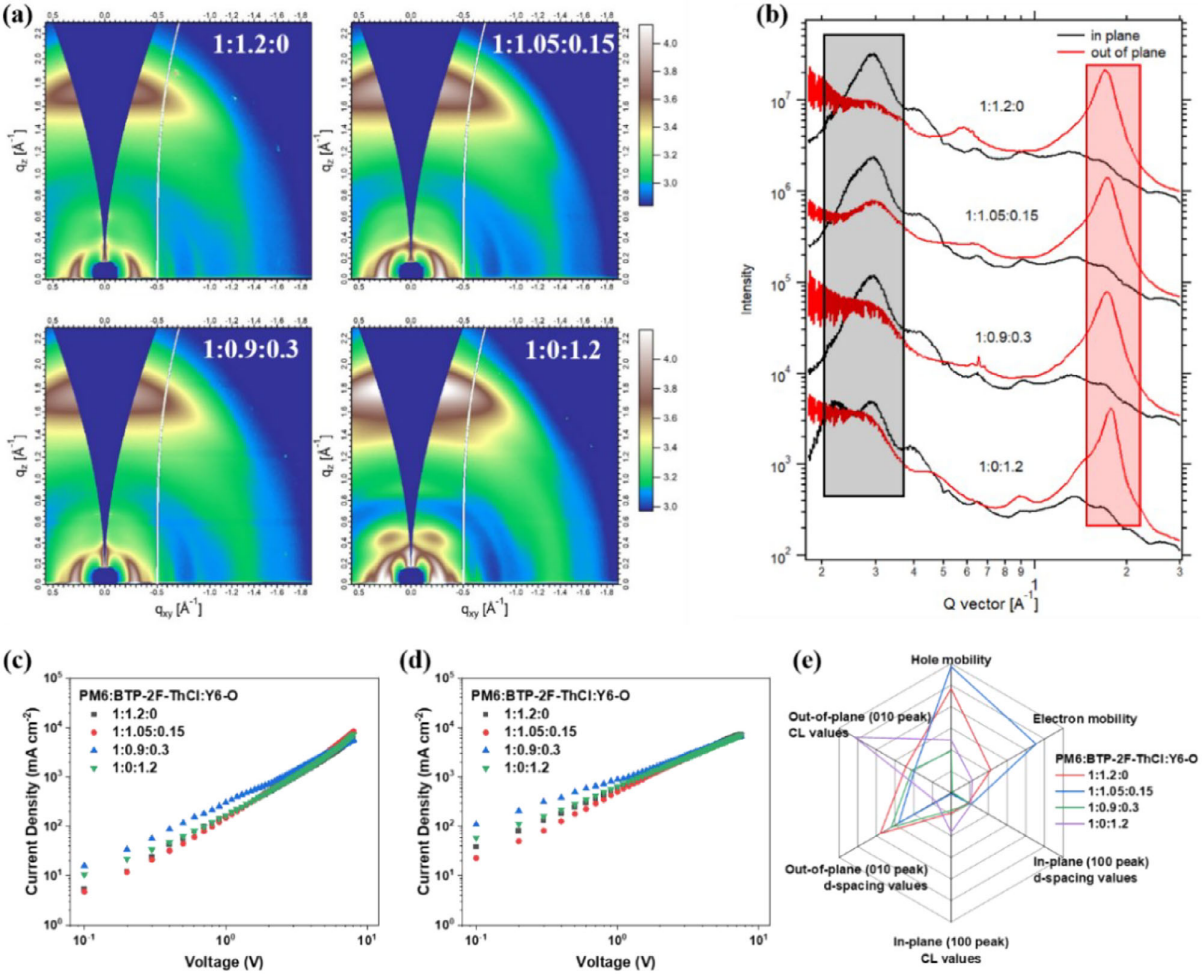
(a) 2D patterns. (b) Line‐cuts. The fitting regions are marked as black and red rectangles, respectively. Results for (c) hole‐only and (d) electron‐only types devices. (e) Correlation visualization for packing parameters and mobilities.

To acquire the morphology information more accurately, the grazing incidence wide‐angle X‐ray scattering (GIWAXS) experiments are implemented [76, 77, 78]. The 2D patterns and corresponding line‐cuts are shown in Figure 2a,b, respectively. All films have strong lamellar signals alongside (in‐plane) IP direction and very significant π–π stacking peaks on the out‐of‐plane (OOP) axis, which means face‐on orientation for them is generally identical. However, the Y6‐O‐based binary film demonstrates something different: On both IP and OPP directions, it has additional peaks. These results indicate that Y6‐O is less miscible with PM6, while BTP‐2F‐ThCl is well miscible with both of them. The co‐crystallization of donor and acceptor materials (herein PM6 and BTP‐2F‐ThCl) can form an interpenetrating nano‐structure with sufficient interfaces that would be beneficial to charge generation. On the contrary, less miscible material combination could result in a less efficient process (for Y6‐O binary devices). Next, we focus on how Y6‐O modulates the general π–π stacking of active layers. With the increase of Y6‐O's content, the *d*‐spacing values of (010) peaks are 3.69, 3.64, 3.66 and 3.70 Å, while the coherence length (CL) values corresponding to them are 22.1, 19.9, 20.5 and 35.8 Å. It tells that Y6‐O's proper incorporation reduces the π–π stacking distances, and maintains the crystallization ordering in a decent manner. Note that Y6‐O is poorly miscible with PM6 as proven, so the analysis upon (010) peaks should exclude the data of its binary film because their π–π peaks are also prone to separate from each other, leading to the fitting useless. Besides, since the *d*‐spacing of Y6‐O is the largest one, but a lower value is gained in the ternary film of 1:1.05:0.15 for PM6:BTP‐2F‐ThCl:Y6‐O, it is supposed to be a guest acceptor insertion case of Y6‐O molecules into PM6:BTP‐2F‐ThCl crystals.

To evaluate the change of charge transport ability in active layers and correlate it to morphology analysis, the hole and electron mobility (*µ*_h_ and *µ*_e_) values are calculated by space charge limited current (SCLC) methods [24, 79]. The hole‐only and electron‐only device results are illustrated in Figure 2c,d, respectively. Consequently, *µ*_h_s of all films are 8.42 × 10^−4^, 8.93 × 10^−4^, 6.97 × 10^−4^ and 7.23 × 10^−4^ cm^2 ^V^−1 ^s^−1^, whereas *µ*_e_s are 5.06 × 10^−4^, 6.27 × 10^−4^, 4.03 × 10^−4^ and 4.57 × 10^−4^ cm^2 ^V^−1 ^s^−1^. The best charge transport property is achieved when PM6:BTP‐2F‐ThCl:Y6‐O ratio is equal to 1:1.05:0.15. This proves that decreased π–π stacking distance contributes to mobility enhancement while slightly lower CL is not very much negative. To further figure out the molecular packing and charge transport formed structure‐property relationship, Figure 2e presents a visual correlation analysis.

**FIGURE 3:**
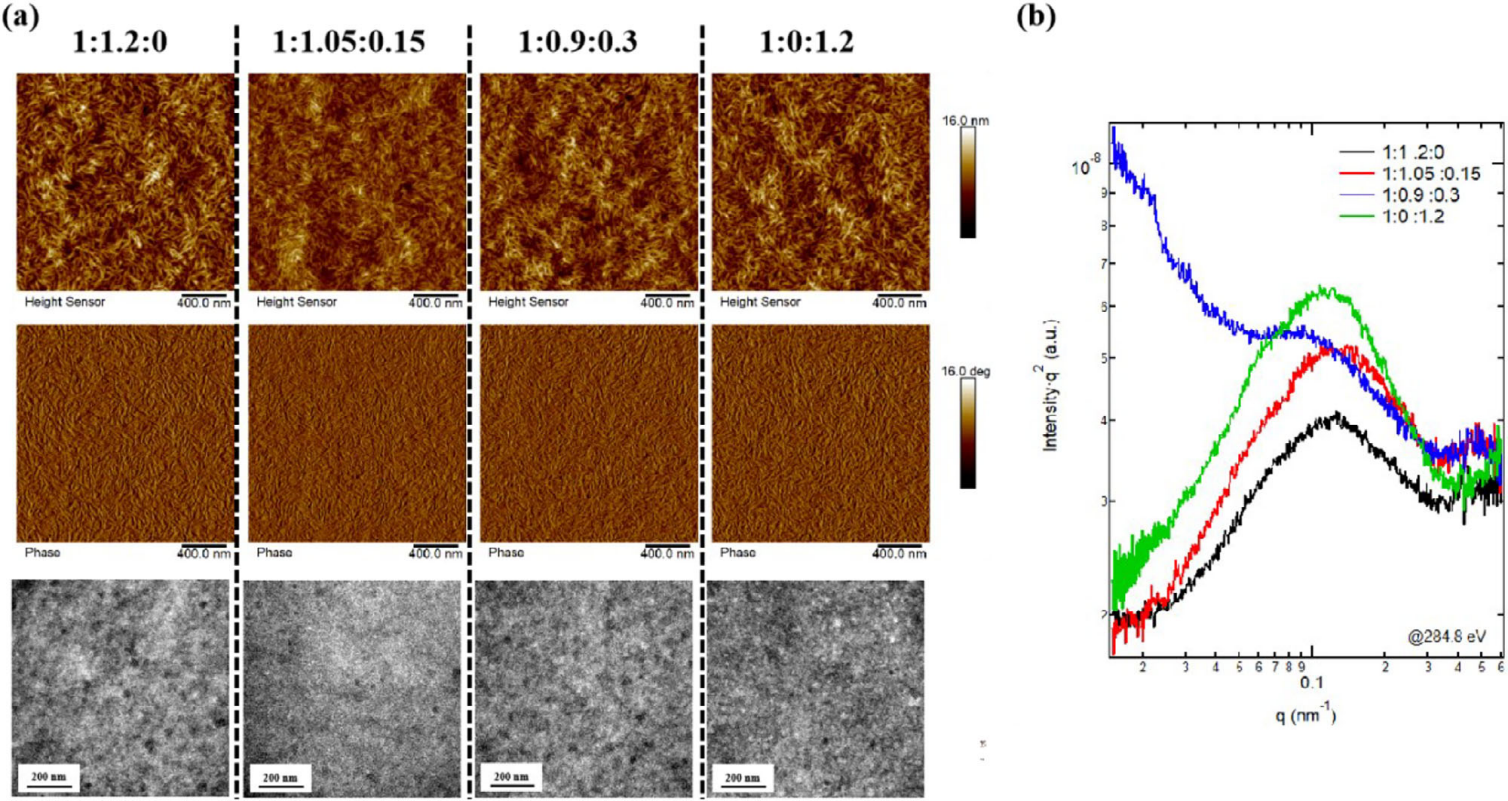
(a) AFM height + phase images and TEM pictures for binary and ternary blend films. (b) RSoXS results.

Then larger scale morphological analysis focusing on phase separation is carried out. The atomic force spectroscopy (Figure 3a) height and phase images, transmission electron spectroscopy photos of marked 200 nm ruler for weight ratio active layers. The root‐mean‐square (Figure 3b) values for them are 2.02, 1.40, 1.78 and 1.87 nm, with the increase of Y6‐O's content. The first reduced and then increased surface roughness tendency suggests a non‐monotonic change for phase separation behavior. The phase images confirm that all films contain favorable nano‐fibrillar network. The TEM results directly show that 1:1.05:0.15 film has suppressed aggregation/separation, compared to other systems no matter the higher or lower Y6‐O proportion. Then the resonant soft X‐ray scattering (RSoXS) technique provides a quantitative illustration of domain size/purity variation [80, 81, 82]. The domain size varies from 28.3, 25.9, 30.0 and 29.1 nm, while the relative purities are 89%, 93%, 97% and 100%. These results demonstrate a reduced domain size and improved domain purity for 1:1.05:0.15 weight ratio system, which is principally beneficial to boosting *J*_SC_ and FF.

After a complete morphology study, the PV performance is investigated by making a series of devices with ITO/PEDOT:PSS‐TA [83] /active layer/ZrAcAc/Ag structure. The results are illustrated as current density versus voltage (*J–V*) characteristics in Figure S1a, and presented as PV parameters in Table S1. The PCE of ternary devices with optimized *J*_SC_ and FF demonstrates an 18.1% value, significantly outperforming that of binary solar cells (17.1%). The device parameter variation is in well consistence with the morphology evolution direction, which proves the effectiveness of this insertion strategy. To assure the measurement is reliable, IPCE spectra of devices are obtained and painted in Figure S1b. Corresponding integrated *J*_SC_ values are placed in Table S1, as well. These results confirm the testing errors are within 2%. Ternary device stability under illumination is evaluated by encapsulating them under 1‐sun LED light soaking. Figure S1e shows the results that the guest acceptor inserted active layer can offer slightly improved photostability. What is more, accompanying device physics data are available to check the charge generation/collection efficiency and recombination status. From Figure S1f–S1g and Table S2, we can conclude the best charge generation and collection, most suppressed trap‐assisted and bimolecular recombination is simultaneously achieved by acceptor inserted ternary PV blend: (i) the saturated current density versus effective voltage (*J*_sat_ vs *V*_eff_) relationships reveal that target system is given an enlarged *J*_sat_, higher charge dissociation (*η*_diss_) and collection (*η*_coll_) rate, suggesting the improved photo capturing and charge extraction ability in the optimal ternary film; (ii) *V*_OC_ versus light intensity semilogarithmic fitting results of ideal factor indicates the least dominant role of trap assisted recombination in target blend; (iii) *J*_SC_ versus light intensity curves of full‐logarithmic demonstration generates the *S* values (fitted slopes) exhibit the largest one of 0.983 for optimal system, which is very close to 1, implying best suppressed bimolecular recombination. Besides, the existence of strong energy transfer is expelled and charge transfer is observed according to the results of photoluminescence (PL) and pure acceptor‐based device data in Figure S1c,d. Also, from the time‐resolved photoluminescence (TRPL, Figure S11) spectra, it can be seen that trace doping of Y6‐O in the binary system possesses a longer decay lifetime.

**FIGURE 4:**
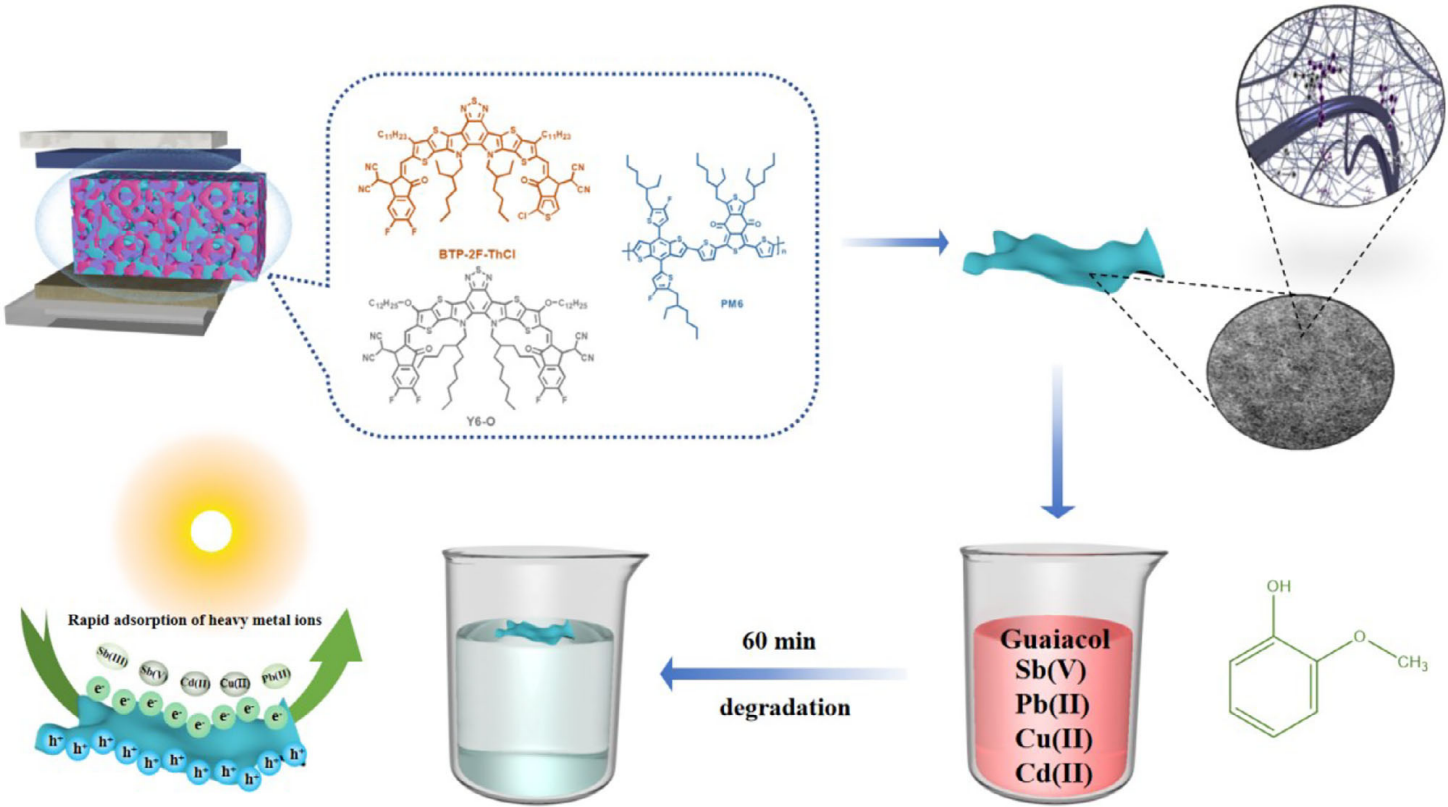
Photocatalytic reaction process.

**FIGURE 5:**
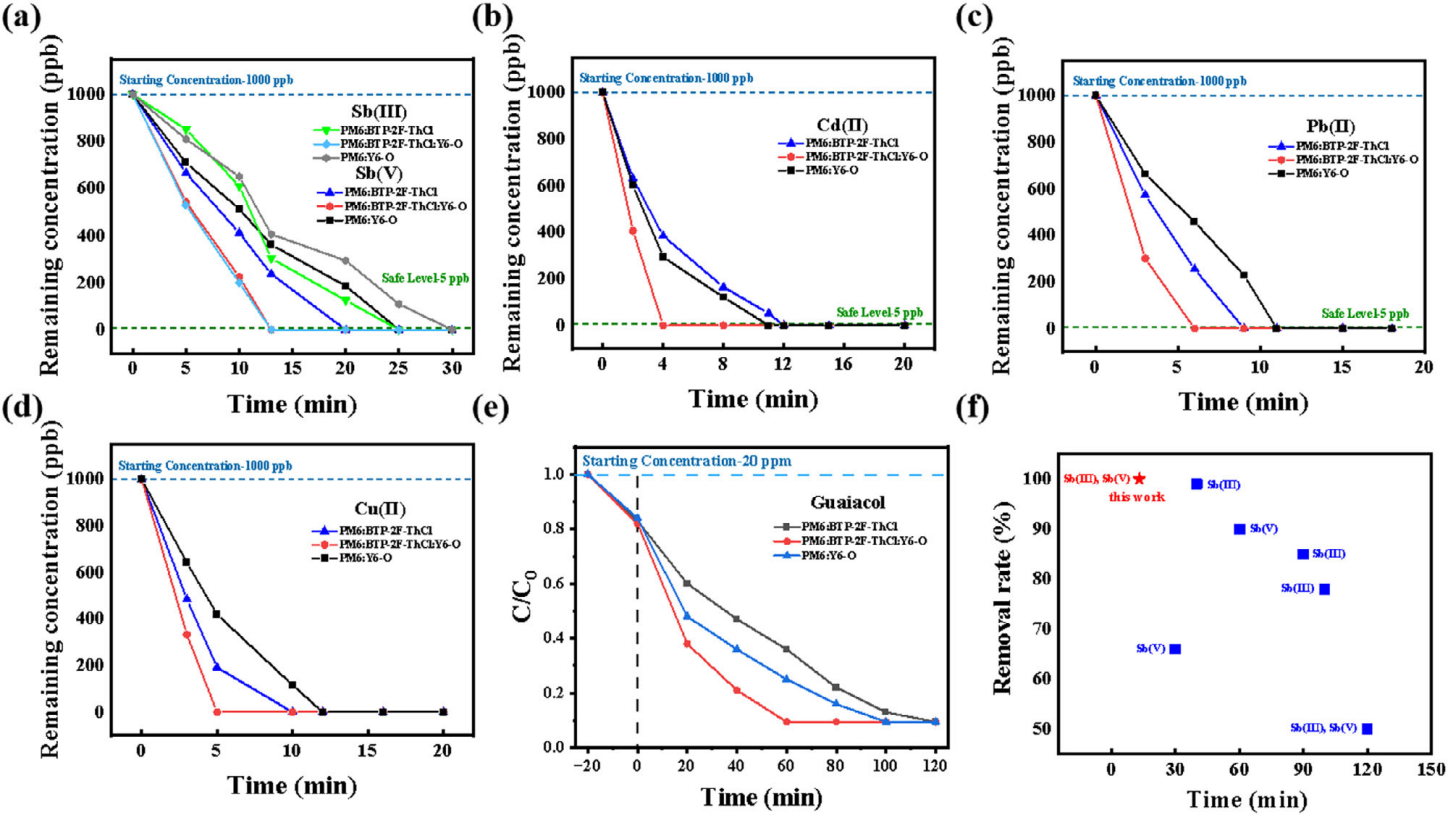
(a) The Sb(III) and Sb(V) reduction contributed by binary and ternary photovoltaic blend‐based membranes. (b) The Cd(II) reduction contributed by binary and ternary photovoltaic blend‐based membranes. (c) The Pb(II) reduction contributed by binary and ternary photovoltaic blend‐based membranes. (d) The Cu(II) reduction contributed by binary and ternary photovoltaic blend‐based membranes. (e) The lignin model compound (guaiacol) degradation contributed by binary and ternary photovoltaic blend‐based membranes. (f) Performance comparison.

Meanwhile, we applied the active layer to the wastewater purification membrane, serving dually as a photo‐adsorbent and photocatalyst, both of which have made significant progress in the removal of heavy metal ions and photorefractory organic pollutants from water. The prepared novel photocatalyst possessed a high specific surface area (1266 m^2^ g^−1^, Figure S15), and its photocatalytic process is shown in Figure 4. The photovoltaic‐derived membranes constructed in this work showed good removal of heavy metal ions Sb(III), Sb(V), Pb(II), Cu(II), and Cd(II) under xenon lamps, as shown in Figure 5. The removal effect of photocatalysts on multiple pollutants under different light conditions is demonstrated in Figures S12 and S13. Notably, the Sb removal performance by our PV‐derived membrane is among the highest levels to date, the comparison with other reports is depicted in Figure 5f, with the details summarized in Table S3. This is a new breakthrough and attempts to use ternary blended membranes as photocatalytic materials to oxidize the difficult‐to‐removal Sb(III) in water to the easily adsorbed Sb(V), which efficiently enhances the purification efficiency of heavy metal ions in water. The results of the subsequent five‐time cyclic removal test of every aforementioned heavy metal ion are given in Figure S2. The membrane removal efficiency of the ternary organic photovoltaic material exceeded 95% and remained stable, which indicates the excellent reusability of the material. In addition, the photovoltaic membranes showed stable and efficient degradation of lignin (guaiacol in paper wastewater). Under dark conditions for 20 min, the bagasse cellulose particle substrate could adsorb 15%–17% of guaiacol. The photodegradation efficiency of guaiacol was 90% within 1 h and remained at 82%–90% photodegradation efficiency after 100 cycles, as detailed in Figure 5e and Figure S3. Figure S16 shows the total organic carbon (TOC) spectra of guaiacol and the results indicate that 98% mineralisation of guaiacol can be achieved in 100 min. In addition, similar high degradation efficiencies of ∼90% were achieved for different weight ratios of 1:1.2:0 and 1:0:1.2 PV‐derived films, as shown in Figure S4. The degradation pathway of guaiacol is demonstrated in Figures S5 and S6. Figure S14 also expresses the removal of phenol, m‐phenol and eugenol by the photocatalyst. Meanwhile, Figure S5 demonstrates the rate constants of PM6:BTP‐2F‐ThCl:Y6‐O for the removal of different pollutants [84, 85].

**FIGURE 6:**
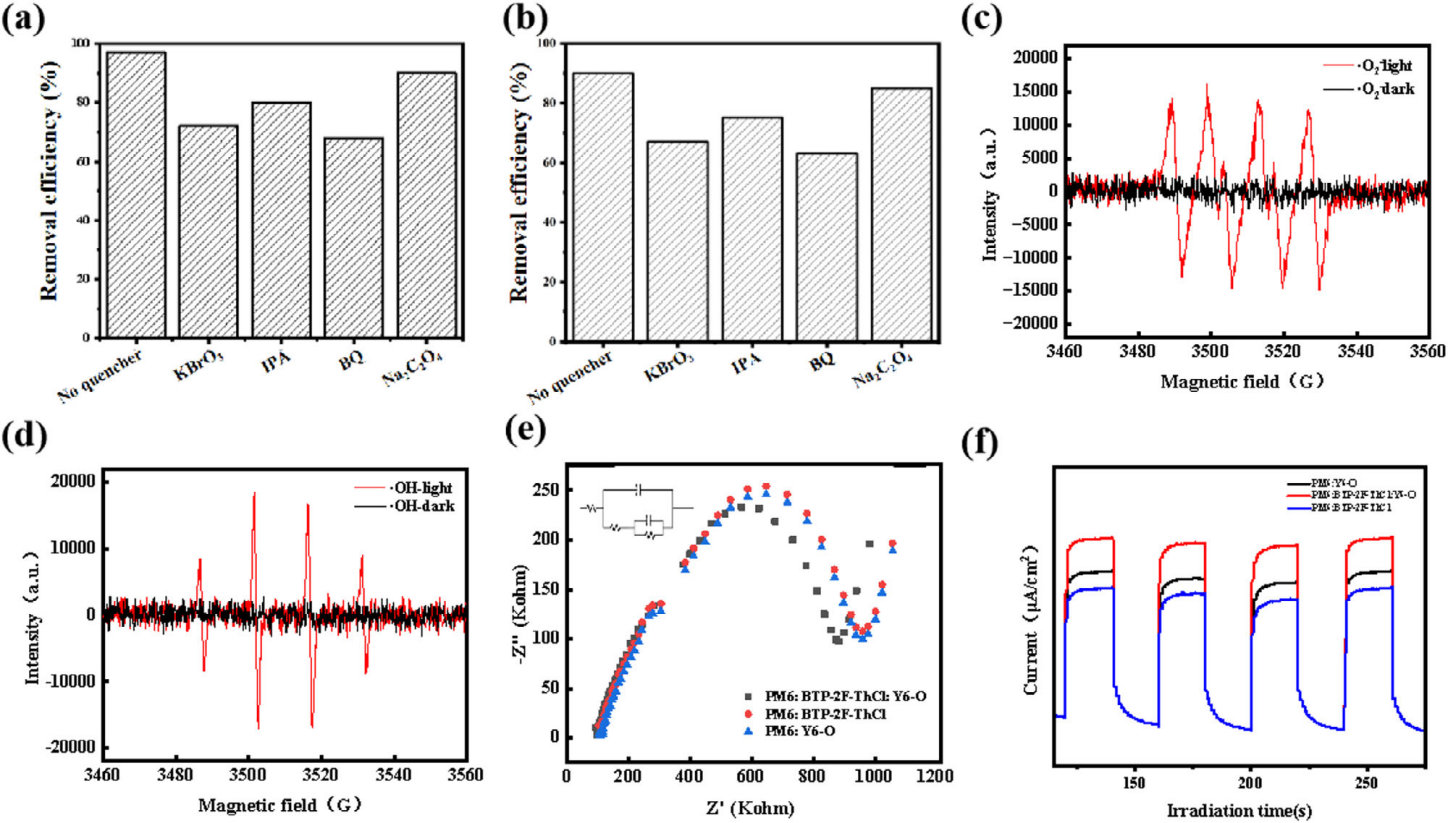
(a) Effects on guaiacol degradation over PM6:BTP‐2F‐ThCl:Y6‐O with different scavengers. (b) Effects on Sb(III) and Sb(V) removal over PM6:BTP‐2F‐ThCl:Y6‐O with different scavengers. The ESR spectra of (c) *·*OH and (d) ·O_2_^−^, (e) EIS Nyquist plots PM6:Y6‐O, PM6:BTP‐2F‐ThCl and PM6:BTP‐2F‐ThCl:Y6‐O, and (f) transient photocurrent responses spectra.

In order to explore and dig deeper into the core active substances for pollutant removal by ternary PV films (PM6:BTP‐2F‐ThCl:Y6‐O), we conducted radical capture experiments and ESR tests. In the radical trapping experiments, Na_2_C_2_O_4_, KBrO_3_, BQ and IPA were used as trapping agents for h^+^, e^−^, ·O_2_^−^ and ·OH as illustrated in Figure 6a,b. The addition of capture agents resulted in significant changes in the effectiveness of ternary photovoltaic films in removing contaminants, for example, the degradation rate of guaiacol decreased from 90% to 62% after the addition of IPA. The removal of Sb(III) and Sb(V) decreased moderately from 100% to 78% with the addition of BQ. The removal rates of guaiacol, Sb(III) and Sb(V) also reduced slightly after the addition of KBrO_3_ and Na_2_C_2_O_4_. The results indicated that ·OH and ·O_2_^−^ played a key role in the removal of Sb(III), Sb(V), Pb(II), Cu(II), Cd(II), and guaiacol. In addition, to further illustrate and determine whether ·O_2_^−^ and ·OH were generated during PM6:BTP‐2F‐ThCl:Y6‐O photocatalysis, ESR tests were performed on ternary photovoltaic films. We utilized 5,5‐dimethyl‐1‐pyrroline *n*‐oxide (DMPO) as a trapping agent. As shown in Figure 6c,d, PM6:BTP‐2F‐ThCl:Y6‐O had strong ·OH and ·O_2_^−^ signals, indicating that the photogenerated electrons in the PM6:BTP‐2F‐ThCl:Y6‐O film has sufficient redox ability to reduce O_2_ to ·O_2_^−^. Meanwhile, the results show that the photogenerated e‐ and h^+^ can be separated effectively in the ternary photovoltaic film (PM6:BTP‐2F‐ThCl:Y6‐O). ESR tests verified that the ternary photovoltaic film produced ·O_2_^−^ and ·OH during the photocatalytic process, and the results are consistent with the active substance capture experiments. Also, we performed EIS spectroscopy (Figure 6e) tests, which were used to further investigate the photogenerated carrier separation and transfer behavior. The charge transfer resistance of PM6:BTP‐2F‐ThCl:Y6‐O was the lowest compared to other binary catalysts, which implied that the PM6:BTP‐2F‐ThCl:Y6‐O photocatalyst exhibited the fastest electron transfer rate. According to the analysis in Figure S1, PM6:BTP‐2F‐ThCl:Y6‐O has a wide range of light absorption (especially in the visible band), fast photogenerated electron‐hole pair separation efficiency and carrier migration efficiency. Figure 6f showed the periodic transient photocurrent response plots of PM6:Y6‐O, PM6:BTP‐2F‐ThCl and PM6:BTP‐2F‐ThCl:Y6‐O catalysts. It can be seen from the plots that the photogenerated current density of PM6:BTP‐2F‐ThCl:Y6‐O was significantly higher than that of PM6:Y6‐O and PM6:BTP‐2F‐ThCl, which indicated that the ternary material had an expanded material light absorption range, higher carrier migration efficiency, better stability and performance compared with the binary material.

**FIGURE 7:**
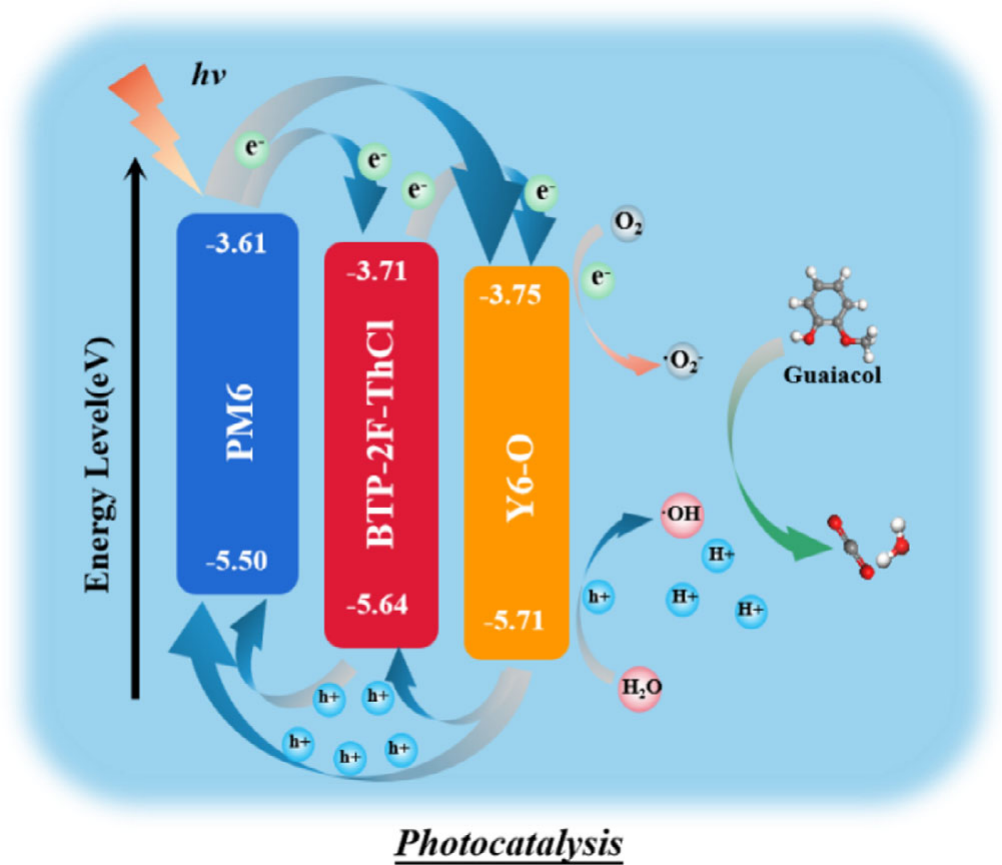
Photocatalytic mechanism diagram.

Figure 7 demonstrates an overall photocatalytic reaction mechanism for the photodegradation of guaiacol by photocatalysts. When the PM6:BTP‐2F‐ThCl:Y6‐O photocatalyst absorbs the light energy from the light source, the valence band electronic conduction band jumps, generating electrons and holes in PM6, BTP‐2F‐ThCl and Y6‐O [74, 75, 86, 87, 88, 89, 90]. Secondly, after the heterojunction formation, the energy band positions of the two semiconductors are rearranged due to the Fermi level equilibrium, that is, the electrons in PM6 migrate to the *E*_LUMO_ of BTP‐2F‐ThCl and Y6‐O, and then the electrons produced by the PYIT also migrate to the *E*_LUMO_ of Y6‐O, whereas the holes produced by light in the *E*_HOMO_ of BTP‐2F‐ThCl and Y6‐O are injected into the *E*_HOMO_ of PM6. In addition, according to the ESR analysis of the PM6:BTP‐2F‐ThCl:Y6‐O photocatalyst, the material produced e^−^, h^+^, ·O_2_^−^ and ·OH under Xe lamp irradiation, and the results showed that *·*O_2_^−^ was produced by e^−^ and O_2_, and ·OH was produced by h^+^ and H_2_O, as shown in the following reaction formula:

(2) $$\text{PM}6:\text{BTP}-2F-\text{ThCl}:Y6-O+\text{hv}\to h^{+}+e$$

(3) $$O_{2}+e^{-}\to \cdot O_{2}^{-}$$

(4) $$H_{2}O+h^{+}\to \text{OH}+H^{+}$$

In addition, we investigated the interactions of PM6, BTP‐2F‐ThCl and Y6‐O by analyzing the PL spectroscopic results and *J*–*V* curves of pure receptor‐based devices. The results observed charge transfer and revealed the presence of strong energy transfer. The effectiveness of charge transfer between the two acceptors suggests that the ternary system has more electron transfer paths than the binary system, and the ternary device has a higher charge transfer efficiency, which may lead to a higher charge transfer efficiency than the binary device. Meanwhile, the ESR test exhibiting the signal results of *·*OH and ·O_2_^−^ generating in the ternary systems are presented in Figure S10 [90, 91].

## 4 Conclusion

We reported the first results of applications integrating the cross‐fields of organic photovoltaics and photocatalysis. Not only has it generated solar cell devices with favorable power conversion efficiency, but also broadened the way of material candidates for advances in environmental remediation. To be specific, we propose an inserting strategy that involves adding a proper amount of guest acceptor based on materials PM6, BTP‐2F‐ThCl, and Y6‐O, guided by the morphology understanding of the organic solar cell (OSC) field. Although Y6‐O has a larger stacking distance, it can be inserted into formed crystals of PM6 and BTP‐2F‐ThCl, resulting in a reduced π–π stacking distance, which improves charge transport in the film. The insertion also reduces the domain size and improves domain purity for the same ratio system, which promotes charge generation and suppresses exciton recombination. This finely tuned morphology in the active layer leads to an efficiency enhancement in ternary OSCs, achieving 18.1% PCE compared to 17.1% or 16.6%. Moreover, we innovatively applied terpolymer organic photovoltaic films to wastewater treatment for the first time in the world, showing exciting removal results with 100% removal in <15 min, achieving the highest removal of ionic Sb(III) and Sb(V) to date, as well as rapid photodegradation of 90% removal of the lignin model compound guaiacol in 1h. Through its pioneering approach, this strategy has provided a much‐needed solution for the removal of heavy metal ions and organic pollutants from the environment.

## Author Contributions

T.L. and L.Y. conceived the idea. L.Y., C.H., Y.Z and Y.L. fabricated devices and finished basic characterizations. L.Y. and Y.Z. performed large‐area device manufacturing and reproduced the claimed results. L.Y., C.H. and T.L. drafted the manuscript. L.Y., B.Z. and T.L. revised the paper. Y.W. and W.M. were in charge of GIWAXS and GISAXS characterizations. L.Y., H.L., Y.Z., T.Y. and C.H. took care of UV, PL and TRPL. L.Z. and H.Z. did heavy metal adsorption and characterization. Y.L., J.X. and S.W. performed photocatalytic analysis and characterization. T.L., W.G., B.Z., T.Y., H.Z., and S.W. gave academic advice. C.H., L.Z. and L.Y. took care of revision. T.L. supervised this project.

## Conflicts of Interest

The authors declare no conflicts of interest.

## Supporting information



Supporting Information

## Data Availability

The data that support the findings of this study are available from the corresponding author upon reasonable request.
