# Supplementary material for: Efficient Ternary Organic Photovoltaic Films for Fast Exciton Separation to Generate Free Radicals for Wastewater Treatment
Source: Exploration (Beijing). 2025 Feb 4;5(3):270001. doi: 10.1002/EXP.70001 (PMC12199427; doi:10.1002/EXP.70001)
Supplement: Supplementary file 1 — Supporting Information [file EXP2-5-270001-s001.docx]

**Supporting Information**

**Efficient Ternary Organic Photovoltaic Films for Fast Exciton Separation to Generate Free Radicals for Wastewater Treatment**

Linji Yang,^a^ Ciyuan Huang,^a,e^ Yang Zhou,^a,e^ Libin Zhang,^a^ Ke Sun,^b^ Houjin Luo,^a^ Yinna Liang,^b^ Yilin Wang,^c^ Tao Yang,^d^ Wei Ma,^c^ Donglou Ren,^a^ Cong Liu,^a^ Heng Zhang,^a^ Kai Chen,^a^ Hongxiang Zhu,^a^ Jianhua Xiong,^a^ Bingsuo Zou,^a^ Shuangfei Wang,^a^ Tao Liu,^a,b*^

1. L. Yang, C. Huang, Y. Zhou, L. Zhang, Y. Liu, Prof. D. Ren, Prof. C.Liu, Prof. H.

Zhang, Prof. K. Chen, Prof. J. Xiong, Prof. B. Zou, Prof. T. Liu,

School of Chemistry and Chemical Engineering, Guangxi Key Laboratory of Processing for Non-ferrous Metals and Featured Materials, State Key Laboratory of Featured Metal Materials and Life-cycle Safety for Composite Structures, School of Resources, Environment and Materials, Guangxi University, Nanning 530004, China.

Email: [liutaozhx@gxu.edu.cn](mailto:liutaozhx@gxu.edu.cn)

1. Prof. K. Sun, Prof. T. Liu,

Youjiang Medical University for Nationalities, Baise 533000, China.

1. Y. Wang, Prof. W. Ma

State Key Laboratory for Mechanical Behavior of Materials, Xi'an Jiaotong University, Xi'an 710049, China.

1. Prof. T. Yang,

Centre for Mechanical Technology and Automation, Department of Mechanical Engineering, University of Aveiro, 3810-193 Aveiro, Portugal.

1. These authors contributed equally.

**Characterizations**

UV-vis absorption spectra were measured using a Shimadzu UV-2500 recording spectrophotometer. TEM was recorded on a JEOL JEM-2100 operated at 120kV. GIWAXS measurements were performed at beamline 7.3.3 at the Advanced Light Source. Samples were prepared on Si substrates using identical blend solutions as those used in devices. The 10 keV X-ray beam was incident at a grazing angle of 0.11°-0.15°, selected to maximize the scattering intensity from the samples. The scattered x-rays were detected using a Dectris Pilatus 2M photon counting detector. RSoXS transmission measurements were performed at beamline 11.0.1.2 at the Advanced Light Source (ALS). Samples for R-SoXS measurements were prepared on a PSS modified Si substrate under the same conditions as those used for device fabrication, and then transferred by floating in water to a 1.5 mm × 1.5 mm, 100 nm thick Si_3_N_4_ membrane supported by a 5 mm × 5 mm, 200 μm thick Si frame (Norcada Inc.). 2-D scattering patterns were collected on an in-vacuum CCD camera (Princeton Instrument PI-MTE). The sample detector distance was calibrated from diffraction peaks of a triblock copolymer poly(isoprene-b-styrene-b-2-vinyl pyridine), which has a known spacing of 391 Å. The beam size at the sample is approximately 100 μm by 200 μm.

**Solar cell fabrication and characterization**

Solar cells were fabricated in a conventional device configuration of ITO/PEDOT:PSS-TA/active layers/ZrAcAc/Ag. The ITO substrates (~92% transmittance) were first scrubbed by detergent and then sonicated with deionized water, acetone and isopropanol subsequently, and dried overnight in an oven. The glass substrates were treated by UV-Ozone for 30 min before use. PEDOT:PSS-TA was spin-cast onto the ITO substrates at 7500 rpm for 30 s, and then dried at 160 °C for 15 min in air. The blend of PM6:acceptors (1:1.2 in weight) blends were dissolved in CF (7 mg·mL^-1^ donor concentration), with 1-CN (0.5% vol) as additive, and stirred on a 50 °C hotplate for 2 hours a nitrogen-filled glove box. The blend solution was spin-cast at 3000 rpm for 50 s onto PEDOT:PSS-TA films followed by a temperature anealing of 100°C for 1 min. ZrAcAc thin layers were coated on the active layer with 3000 rpm (0.5 mg·mL^-1^), followed by the deposition of Ag (150 nm) (evaporated under 1×10^-3^ Pa through a shadow mask). The optimal active layer thickness measured by a Bruker Dektak XT stylus profilometer was about 105 nm. The current density-voltage (J-V) curves of devices were measured using a Keysight B2901A Source Meter in glove box under AM 1.5G (100 mW·cm^-2^) using a Enlitech solar simulator. The device contact area was 0.05 cm^2^, device illuminated area during testing was 0.04 cm^2^, which was determined by a mask. The EQE spectra were measured using a Solar Cell Spectral Response Measurement System QE-R3011 (Enlitech Co., Ltd.). The light intensity at each wavelength was calibrated using a standard monocrystalline Si photovoltaic cell.

**SCLC Measurements**

The electron and hole mobility were measured by using the method of space-charge limited current (SCLC) for electron-only devices with the structure of ITO/ZnO/active layer/ZrAcAc/Ag and hole-only devices with the structure of ITO/PEDOT:PSS-TA/active layers/MoO_x_/Ag. The charge carrier mobility was determined by fitting the dark current to the model of a single carrier SCLC according to the equation: *J* = 9*ε*_0_*ε*_r_*μV^2^*/8*d^3^*, where *J* is the current density, *d* is the film thickness of the active layer, *μ* is the charge carrier mobility, *ε*_r_ is the relative dielectric constant of the transport medium, and *ε*_0_ is the permittivity of free space. *V* = *V*_app_ –*V*_bi_, where *V*_app_ is the applied voltage, *V*_bi_ is the offset voltage. The charge carrier mobility was calculated from the slope of the *J*^1/2^ ~ *V* curves.

**The Analysis of Jph vs Veff relationships**

The definition of *J*_ph_ is the current density under illumination (*J*_L_) minus the dark current density (*J*_D_), and *V*_0_ refers to the voltage value when *J*_ph_ = 0. Accordingly, *V*_eff_ = *V*_0_ - *V*_appl_, where *V*_appl_ represents applied voltage, has a clear meaning. Importantly, when *V*_eff_ reaches a high value (> 2V) it is normally believed that generated excitons are fully collected, in which *J*_ph_ is equal to saturated current density (*J*_sat_). Then, we can calculate *J*_SC_/*J*_sat_ and *J*_max_/*J*_sat_ to describe exciton dissociation (*η*_diss_) and charge collection (*η*_coll_) efficiency. *J*_max_ is the *J*_ph_ at the maximal output point.

**Preparation of water purification membranes**

Preparation of bagasse cellulose granules: 6.0 g of bagasse cellulose was dissolved in zinc chloride solution (72 wt%) and stirred for 30 min at 353 K. After dissolving into a colloidal solution, 10 mL of polyethylene glycol solution (10 wt%) was added and stirred for 30 min at 800 r/min. After dissolved into a colloidal solution, 10 mL of polyethylene glycol solution (10 wt%) was added and stirred for 30 min, then 20.0 g of Na_2_SO_4_ was added as a roughing agent and stirred at 800 r/min for 30 min, then poured into molds, washed with deionized water and freeze-dried to complete the preparation.

**Preparation of photocatalysts**

PM6:BTP-2F-ThCl:Y6-O (weight ratios of 1:1.2:0, 1:1.05:0.15 and 1:0:1.2) was loaded on the surface of bagasse cellulose particles in the ratio of 1:3000 w/w. The loading of N-TiO_2_ was carried out in the same way as for PM6:BTP-2F-ThCl:Y6-O. Once the membrane preparation is compeleted, keep dry and dark storage.


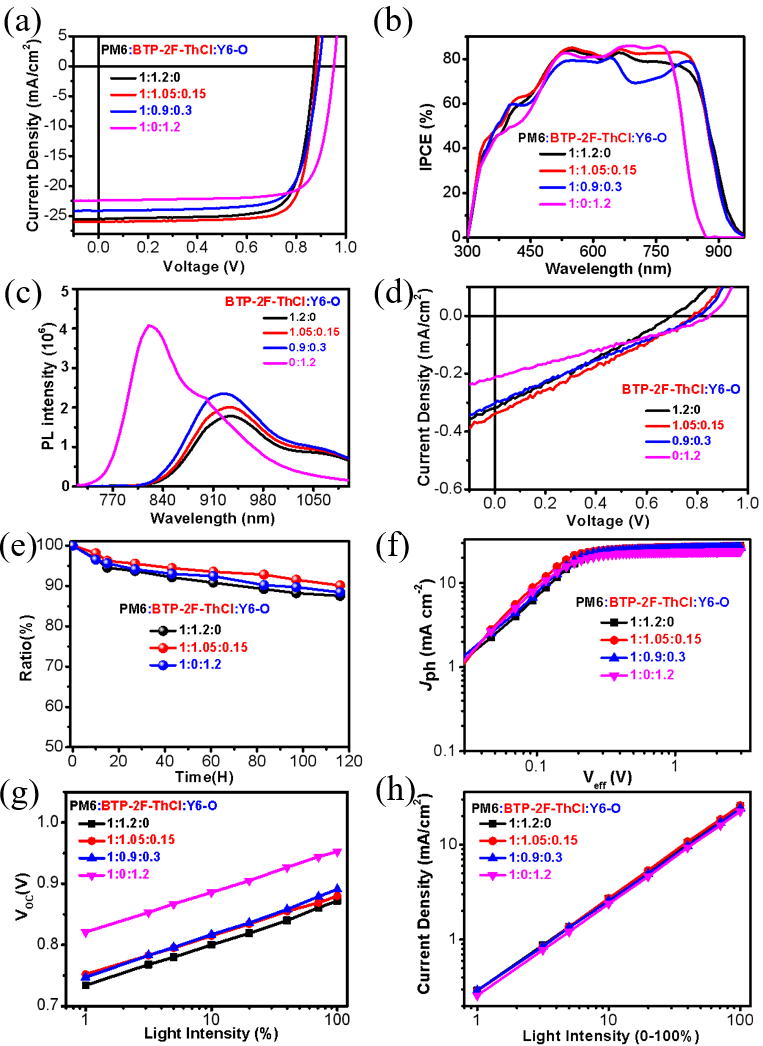


**Figure S1**. (a) *J-V* characteristics. (b) IPCE spectra. (c) PL spectra. (d) Pure-acceptor-based device results.(e) Light-soaking stability. (f) *J*_ph_-*V*_eff_ relationships. (g) *V*_OC_ vs light intensity and (h) *J*_SC_ vs light intensity curves.

**Table S1.** Photovoltaic parameters.

| **PM6:BTP-2F-ThCl:Y6-O** | ***V*_OC_ (V)** | ***J*_SC_ (mA cm^-2^)*^a^*** | ***FF* (%)** | **PCE (%)*^b^*** |
| --- | --- | --- | --- | --- |
| 1:1.2:0 | 0.872 | 25.54/25.11 | 76.6 | 17.1 (16.8±0.2) |
| 1:1.05:0.15 | 0.881 | 26.00/25.72 | 79.0 | 18.1 (17.9±0.1) |
| 1:0.9:0.3 | 0.892 | 24.16/23.99 | 75.5 | 16.3 (16.2±0.2) |
| 1:0:1.2 | 0.952 | 22.46/22.11 | 77.7 | 16.6 (16.5±0.2) |

*^a^*EQE integrated *J*_SC_ values are listed after the slashes. *^b^*The brackets contain averages and standard errors of PCEs based on at least 20 devices.

**Table S2.** Device physics parameters

| Ratio | *J*_sat_ | *J*_SC_ | *J*_max_ | *η*_diss_ | *η*_coll_ | n | *S* |
| --- | --- | --- | --- | --- | --- | --- | --- |
| 1:1.2:0 | 27.53 | 25.54 | 23.08 | 92.7% | 83.8% | 1.14 | 0.972 |
| 1:1.05:0.15 | 27.36 | 26.00 | 24.35 | 95.0% | 89.0% | 1.07 | 0.983 |
| 1:0.9:0.3 | 26.27 | 24.16 | 21.98 | 91.9% | 83.6% | 1.19 | 0.960 |
| 1:0:1.2 | 24.10 | 22.46 | 20.71 | 93.2% | 85.9% | 1.11 | 0.972 |

**TableS3.** Comparison with other materials

| materials | Sb(Ⅲ) initial concentration (μg/L) | Sb(Ⅴ) initial concentration (μg/L) | Sb(Ⅲ) reaction time (min) | Sb(Ⅴ) reaction time (min) | Removal rate (%) | Ref. |
| --- | --- | --- | --- | --- | --- | --- |
| TiO2-in-CNT | 5000 | - | 40 | - | 99 | ^1^ |
| L1M2BO | 50 | 50 | 100 | 30 | 78, 66 | ^2^ |
| FBC | 30 | - | 90 | - | 85 | ^3^ |
| rGO | 10-250 | 10-250 | 120 | 120 | 50 | ^4^ |
| CHBC | - | 40 | - | 60 | 90 | ^5^ |
| This work | 1000 | 1000 | 15 | 13 | 100 | This work |

**The 5-time cyclic removal test of heavy metal ions**


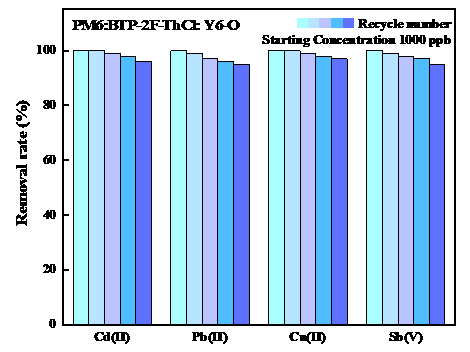


**Figure S2**. The cyclic results of heavy metal ions within 5-time recycle.

**The 100-time cyclic photodegradation results of guaiacol**

To verify the stability and reusability of the PV-derived membranes whose weight ratio is 1:0.5:0.15 when applied to degrade guaiacol, a 100-time cyclic photodegradation experiment is carried out and the details are shown in **Figure. S2**. The photodegradation efficiency maintains 82~90%, implying the PV-derived membranes’ prominent properties.


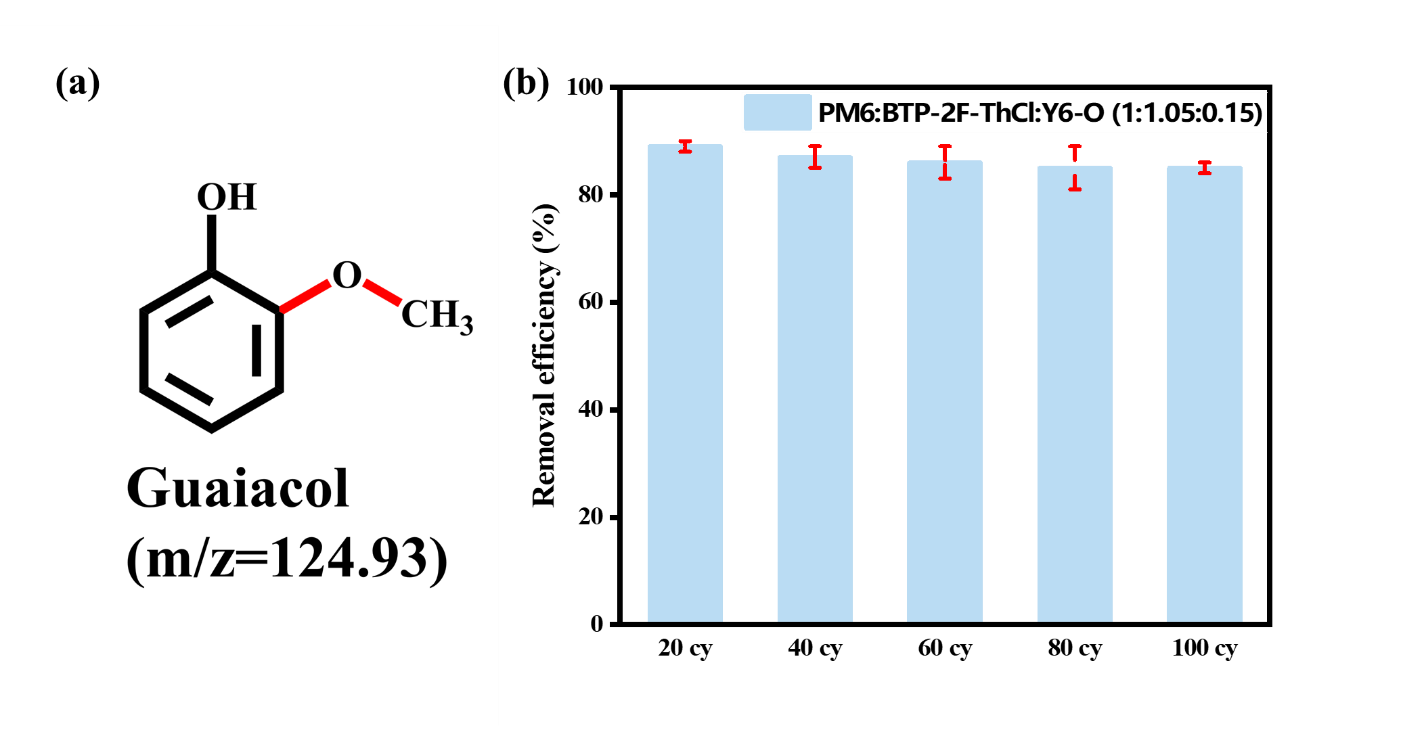


**Figure S3**. (a) Chemical structures of guaiacol; (b) The photodegradation results of guaiacol within 100-time recycle.

**Comparisons of guaiacol photodegradation using ternary organic photocatalyst and traditional inorganic photocatalysts**

**Figure S4**. Comparisons between ternary organic photocatalyst and traditional inorganic photocatalysts.


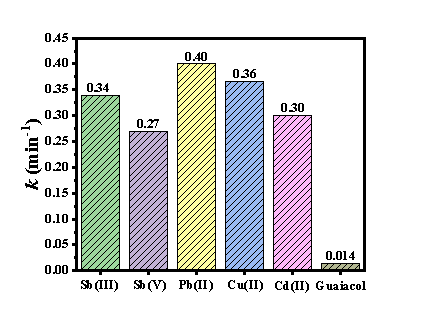


**Figure S5.** PM6:BTP-2F-ThCl:Y6-O rate constant plots for different pollutants.

The chemical structure of guaiacol is shown in **Fig. S6a**. It is worth noting that the main body of the guaiacol molecule is a benzene ring structure, which eliminates the need to consider the atoms located at the end of the benzene ring due to its low reactivity. The HOMO and LUMO orbitals of guaiacol are predominantly clustered around the C1, C2, C4, O8, and O9 atoms, indicating that these atoms are more susceptible to attack, as shown in **Fig. S6b** and **Fig. S6c**. As shown in **Fig. S6d**, the electrostatic surface potential (ESP) of guaiacol represents the dispersion of the surface electron cloud. It is clear that negatively charged oxygen atoms are more inclined to undergo electron-loss reactions in branched structures. However, neither HOMO nor LUMO can accurately and quantitatively characterize the reactivity of each site, so the Fukui index, which represents the electrophilic (*f* ^-^), nucleophilic (*f* ^+^), and radical attack (*f*_0_) of the atoms on the guaiacol molecule, was computed and is presented in **Fig. S7**. This approach can further elucidate the attack of ROS on guaiacol molecules and help to accurately predict the reaction sites for radical attraction. In general, atoms with higher *f* ^-^, *f* ^+^ and *f*_0_ values are more susceptible to attack by *h^+^*, *·O_2_^-^* and *·OH*, respectively. Therefore, the reaction sites of guaiacol are located at C1, C2, C4, O8 and O9, since their Fukui indexes are much higher than the other atoms, which suggests that the C1, C2, C4, O8 and O9 atoms are the main sites of radical attack, which is in better agreement with the degradation pathways 1, 2 and 3, respectively (**Fig. S9**).


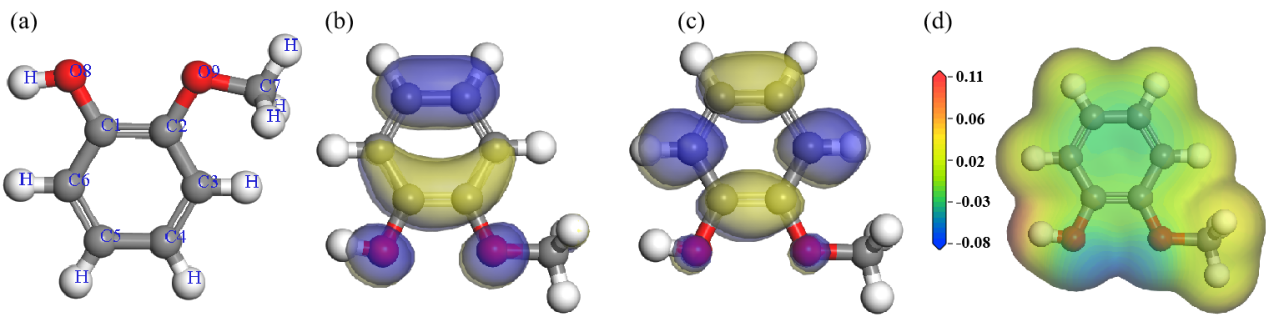


**Fig. S6**. DFT calculations on guaiacol molecule: (a) guaiacol chemical structure; (b) HOMO and (c) LUMO orbital distributions of guaiacol and (d) ESP.

| No. | Atom | *f* ^-^ | *f* ^+^ | *f* ^0^ |
| --- | --- | --- | --- | --- |
| 1 | C | 0.061 | 0.055 | 0.058 |
| 2 | C | 0.061 | 0.053 | 0.057 |
| 3 | C | 0.03 | 0.119 | 0.074 |
| 4 | C | 0.053 | 0.031 | 0.042 |
| 5 | C | 0.052 | 0.023 | 0.037 |
| 6 | C | 0.031 | 0.123 | 0.077 |
| 7 | C | 0.047 | 0.041 | 0.044 |
| 8 | O | 0.109 | 0.060 | 0.085 |
| 9 | O | 0.093 | 0.038 | 0.065 |


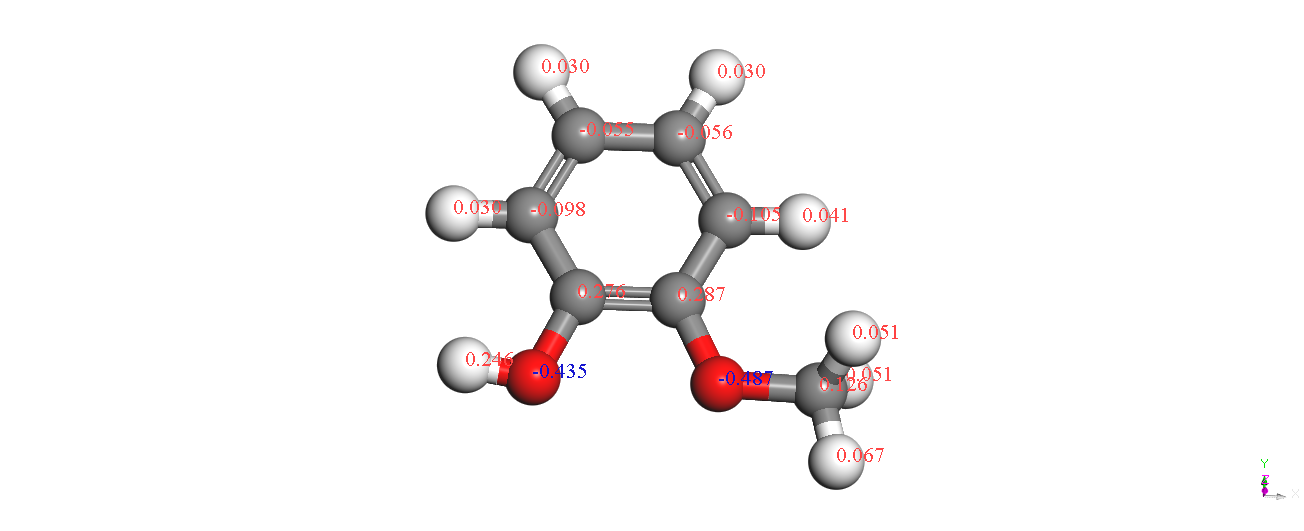


**Fig. S7**. Surface electrostatic potential and Fukui index of guaiacol.

To better understand the reactive oxygen species-induced degradation pathway of guaiacol, the mass spectra of the intermediates are shown in **Fig. S8** and a possible degradation pathway is hypothesized in **Fig. S9**. The degradation intermediates of guaiacol were identified using LC-MS (**Fig. S8**). The three main degradation pathways are depicted in **Fig. S9**. The degradation intermediates of guaiacol were identified using LC-MS (**Fig. S9**). The product m/z = 124.93 represents the molecular weight of guaiacol. Path 1: As illustrated in **Fig. S9**, guaiacol is transformed into P1 (m/z= 110.96), P4 (m/z= 125.10), and P6 (m/z= 124.04). Under the effect of free radicals (mostly ·OH), the methoxy group of guaiacol is replaced by a radial group, resulting in the P1 product catechol. Moreover, since the hydroxyl group is an ortho-positioning group, electrophilic substitution primarily occurs in the ortho-position of the hydroxyl group. Specifically, the ortho-position of the hydroxyl group of the catechol is attacked by free radicals, resulting in P4 pyrogallol and P6 (m/z=124.04).(Kermani et al., 2018) Path 2: The major product, P2 p-benzoquinone, is produced when *·OH* directly attacks the methoxy functional group removed by CH_3_-. Path 3: Phenol is the result of the P3 pathway, which involves free radicals attacking the methoxy group of guaiacol. H_2_O then attacked the aromatic ring of phenol, causing it to dehydrogenate into catechol P5. Electrophilic radicals first attacked the ring to create cationic radicals.(Zeng et al., 2015) Then, P5 can be dehydrooxidized to form o-quinone P7.(Xing et al., 2023) The unstable quinones created by pathway 1, 2, and 3 are then further oxidized by free radicals into tiny molecular acids such oxalic acid, acetic acid, and formic acid, which are then oxidized and destroyed into CO_2_ and H_2_O.


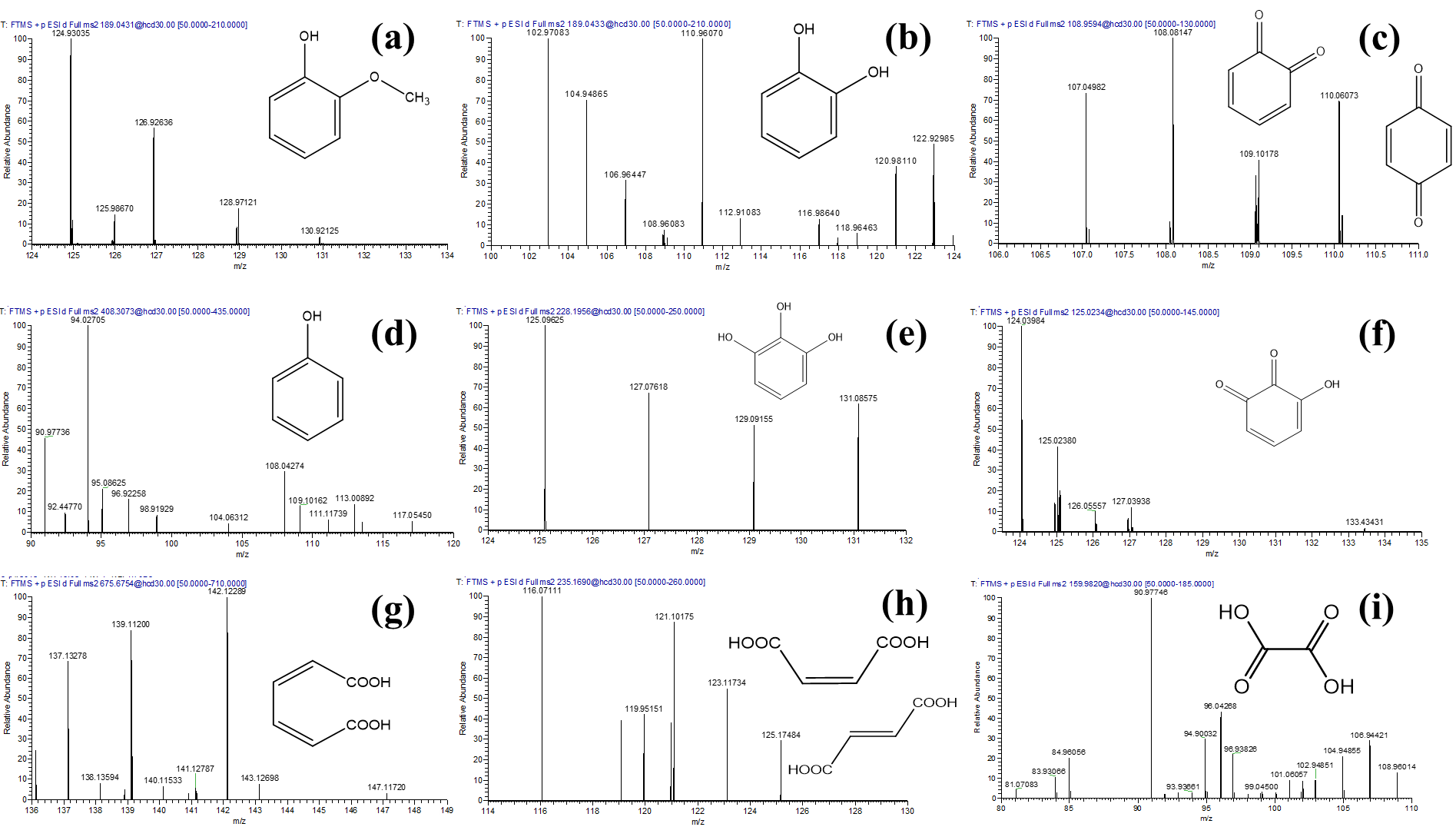


**Fig. S8.** The mass spectrum of the identified intermediates of guaiacol degradation.


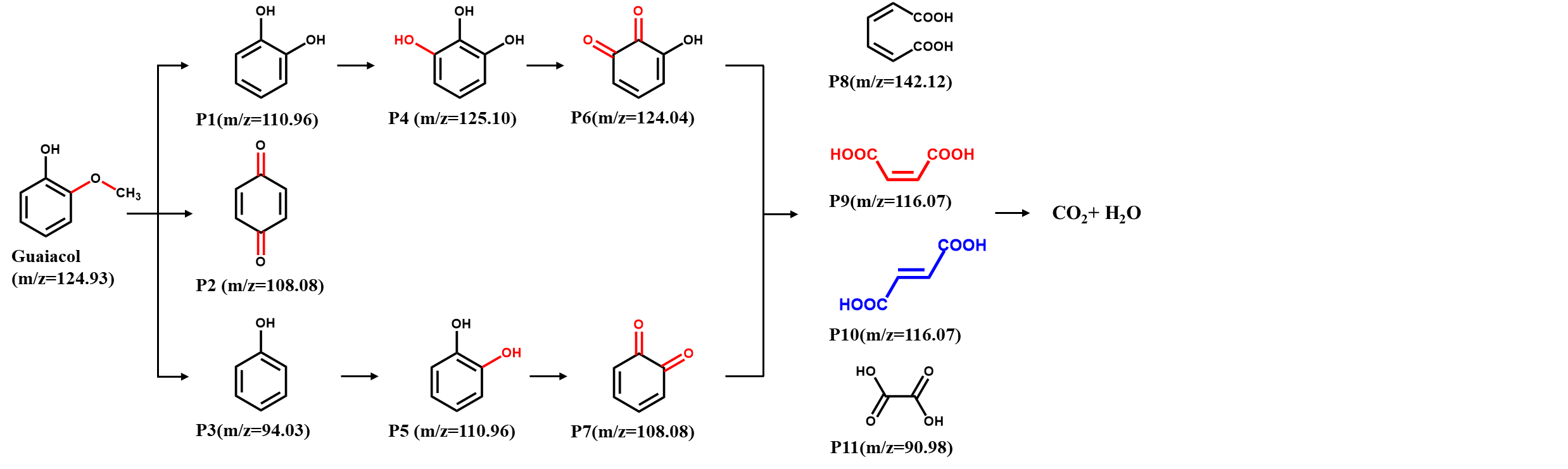


**Fig. S9.** Possible photocatalytic degradation pathways of guaiacol.


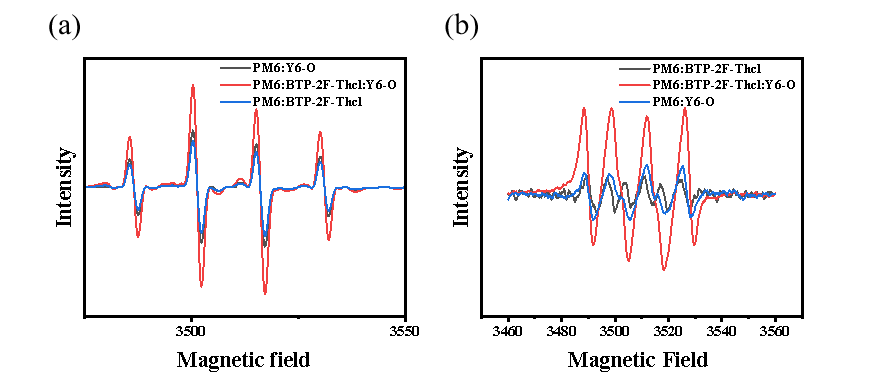


**Figure S10.** The ESR spectra for different catalysts (a) ·OH and (d) ·O_2_^-^.


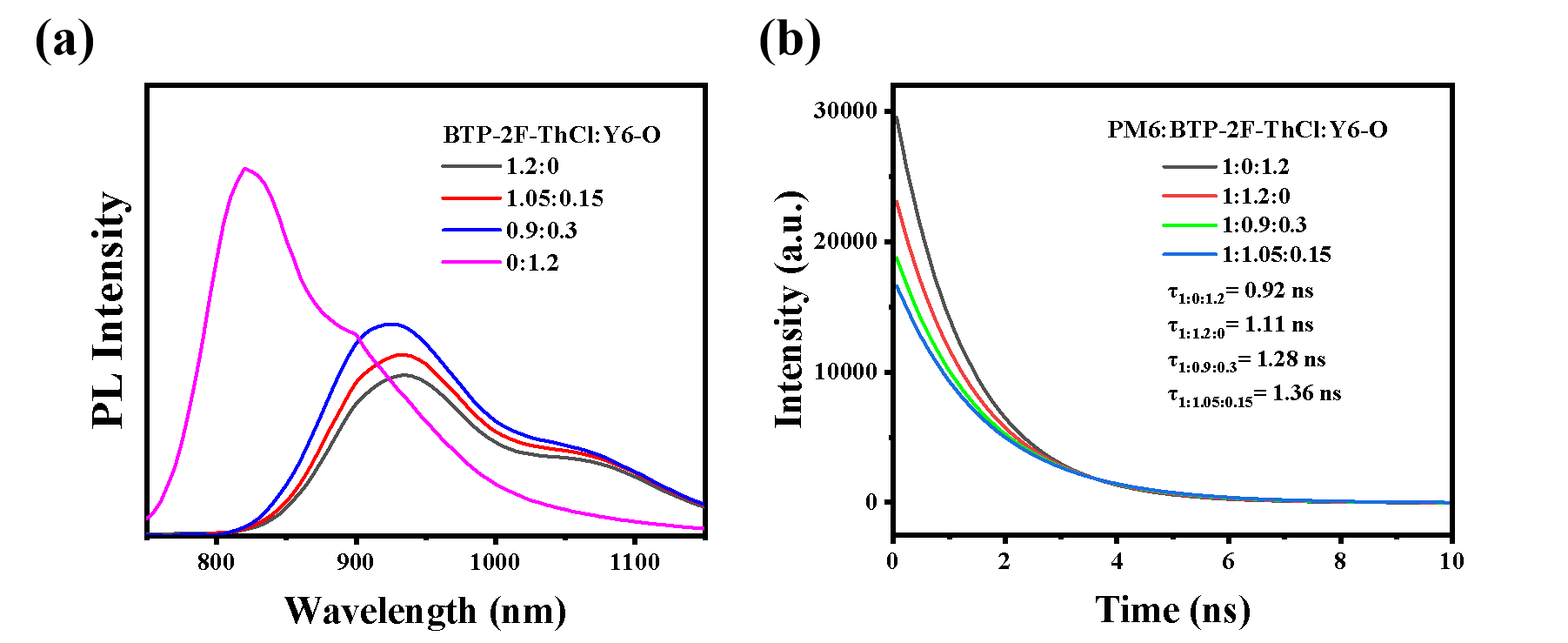


**Figure S11.** (a) Photoluminescence (PL) spectra of BTP-2F-ThCl:Y6-O, (b)Time-resolved photoluminescence (TRPL) spectra of PM6:BTP-2F-ThCl:Y6-O.


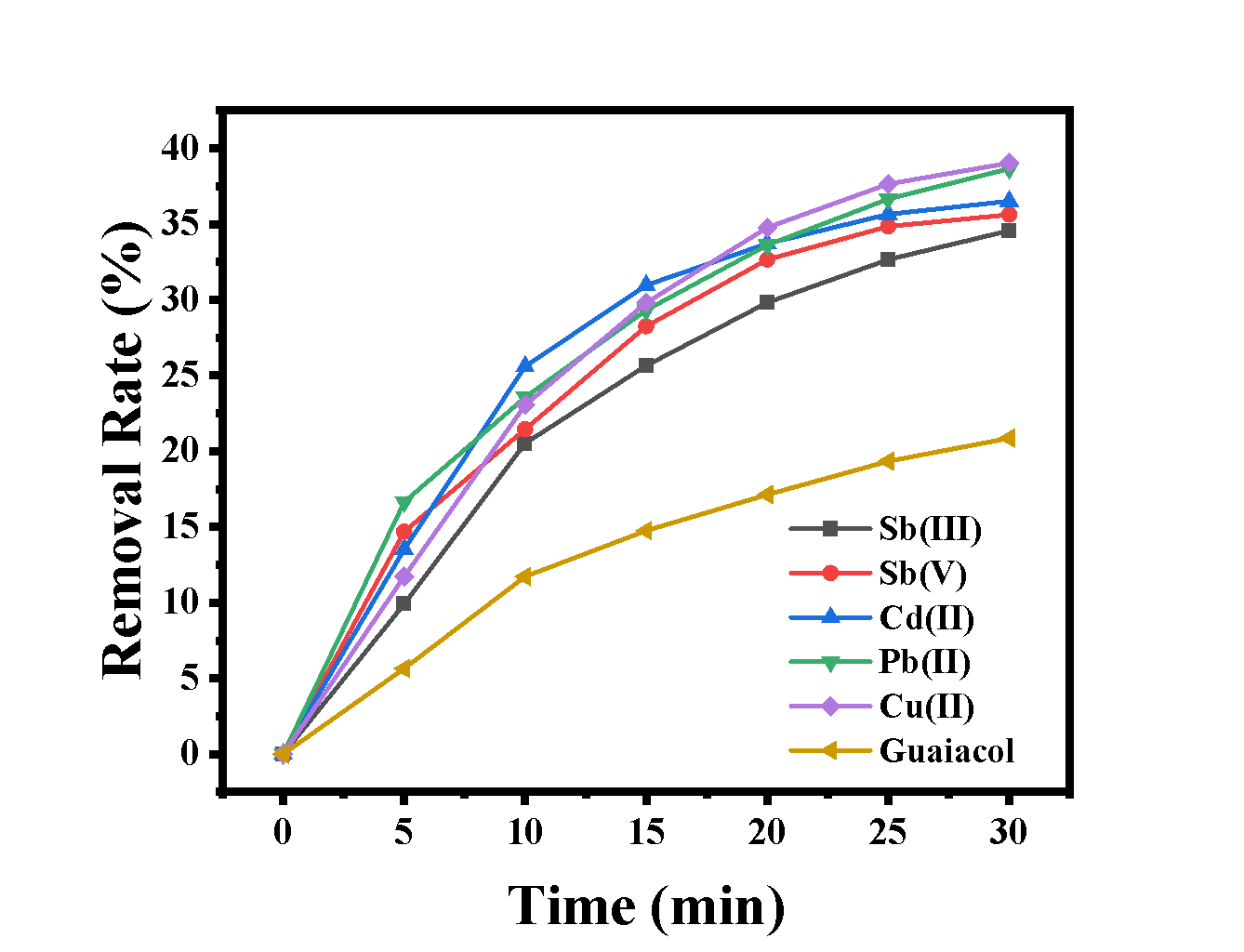


**Figure S12.** Removal of Sb(Ⅲ), Sb(Ⅴ), Cd(Ⅱ), Pb(Ⅱ), Cu(Ⅱ), guaiacol by ternary photovoltaic hybrid membranes under dark conditions. (Sb(Ⅲ), Sb(Ⅴ), Cd(Ⅱ), Pb(Ⅱ), Cu(Ⅱ) were all at a concentration of 1 mg/L, guaiacol was at a concentration of 20 mg/L, and the catalyst was PM6:BTP-2F-ThCl:Y6-O)


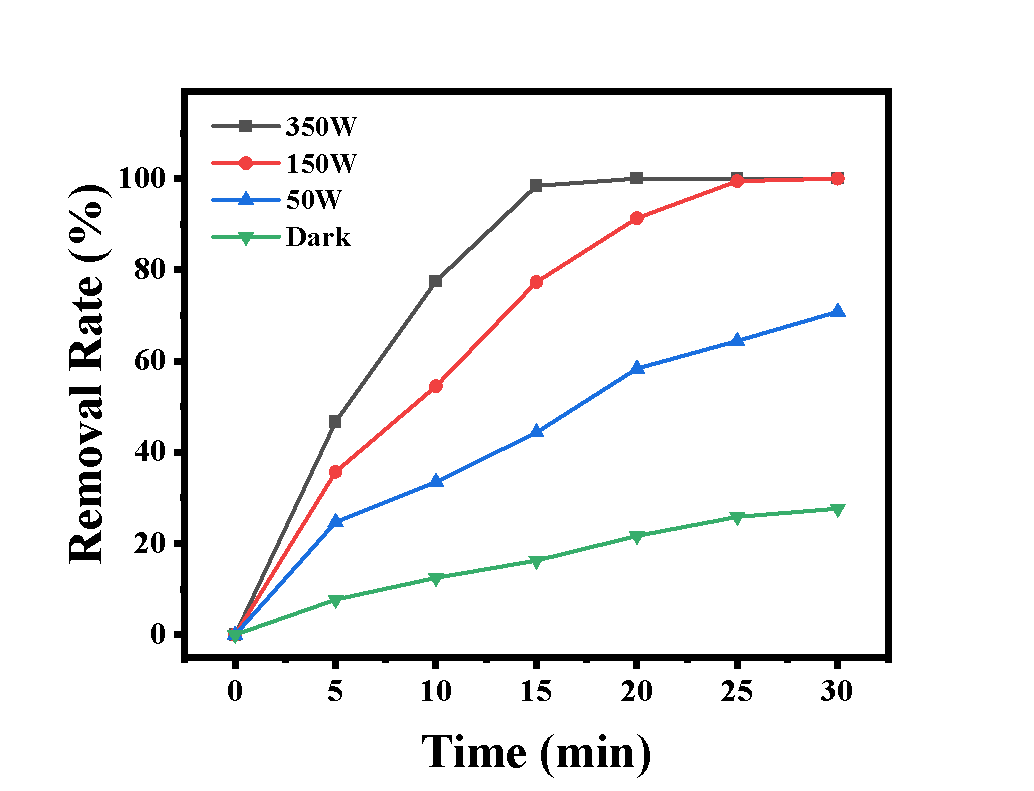


**Figure S13.** Removal of Sb(V) at different light intensities. (The starting concentrations of Sb(V) were all 1 mg/L, and the catalyst was PM6:BTP-2F-ThCl:Y6-O)


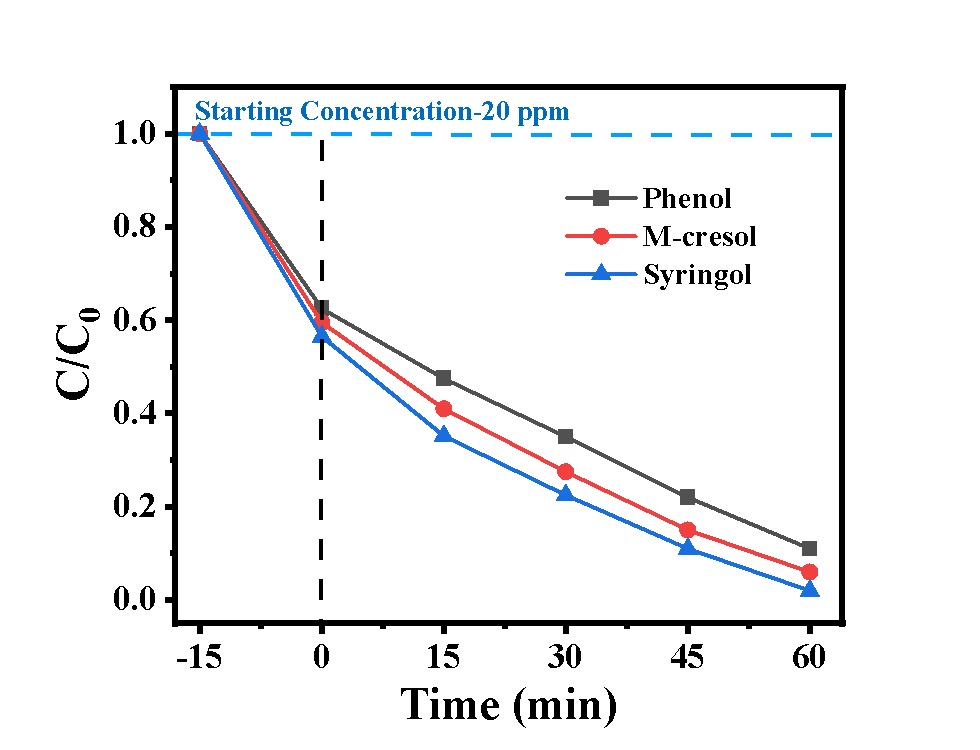


**Figure S14.** Removal effect of PM6:BTP-2F-ThCl:Y6-O on phenol, m-phenol, and syringol.


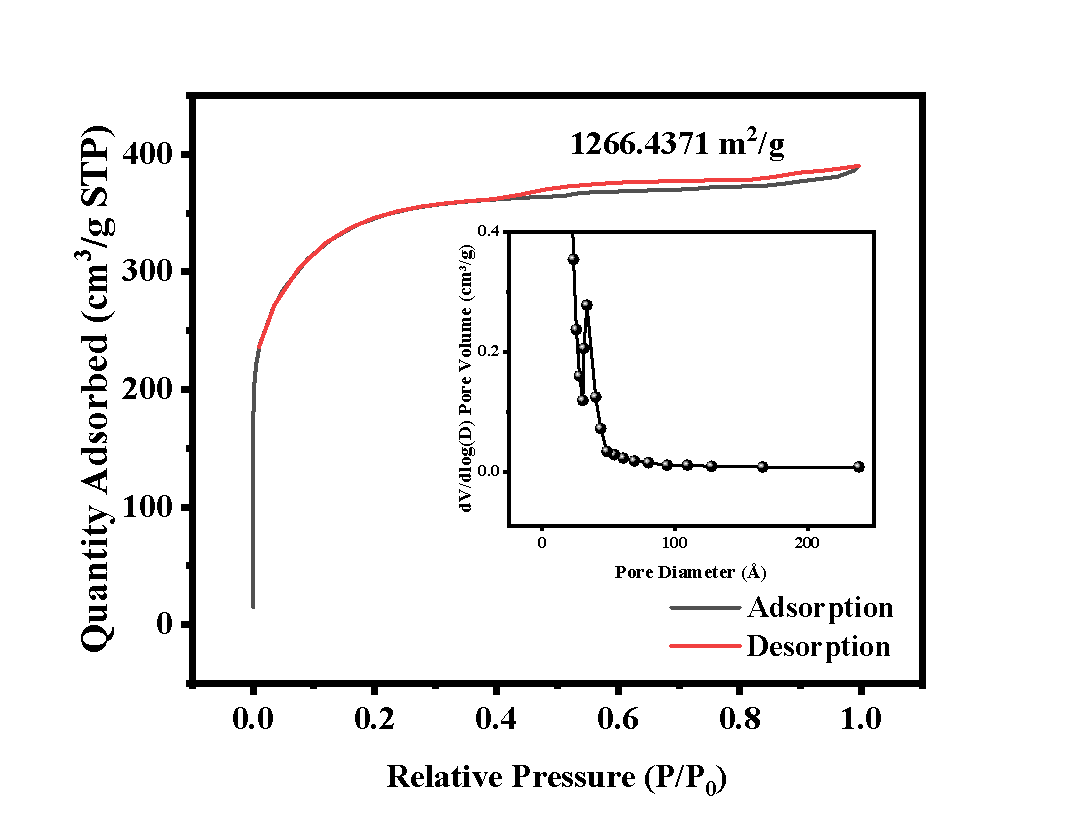


**Figure S15.** (a) Nitrogen adsorption-desorption isotherms and corresponding pore sizes for PM6:BTP-2F-ThCl:Y6-O.


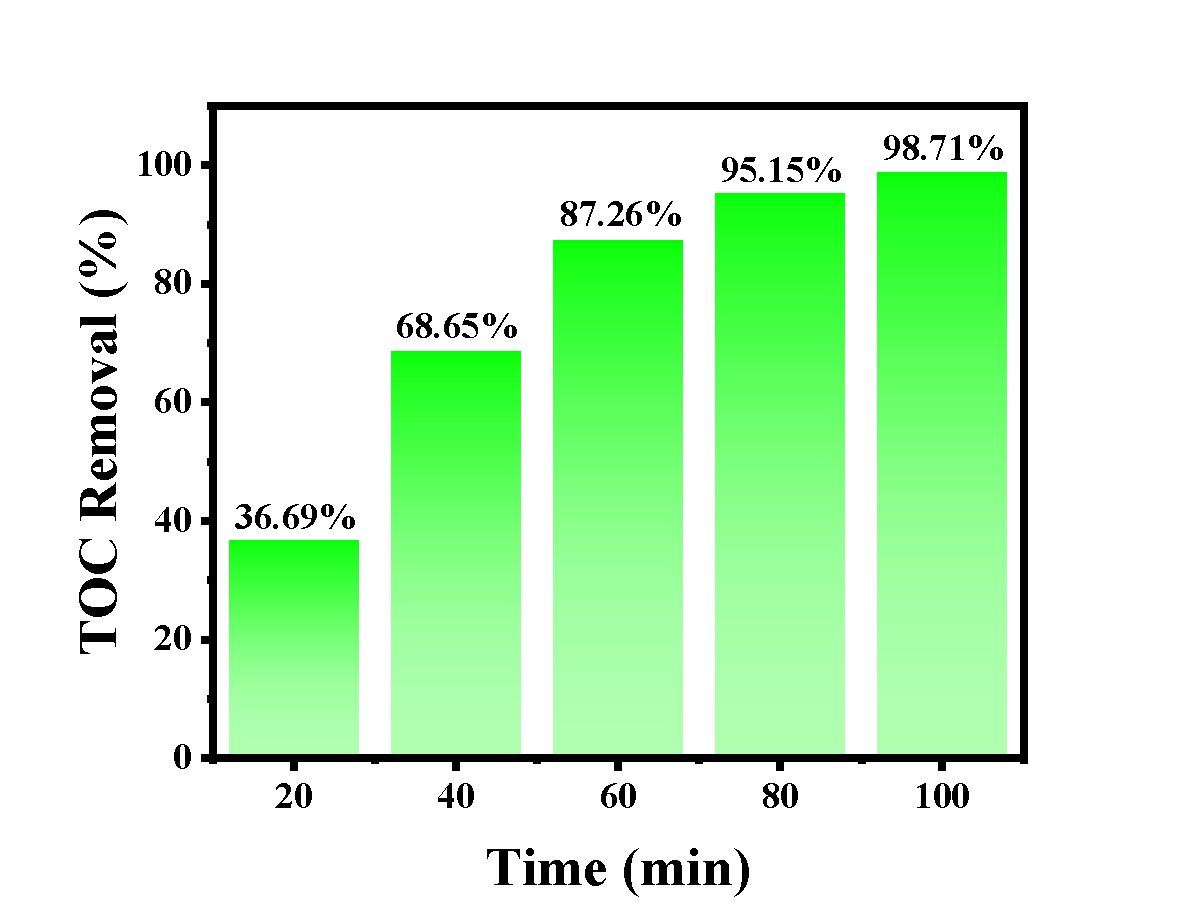


**Figure S16.** TOC characterization.

1. Y. Ren, D. Guo, Z. Zhao, P. Chen, F. Li, J. Yao, H. Jiang and Y. Liu, *Chemical Engineering Journal*, 2022, **435**, 134832.

2. C. Zhang, M. Wu, K. Wu, H. Li and G. Zhang, *Journal of Hazardous Materials*, 2023, **442**, 130132.

3. L. Zhang, Y. Dong, J. Liu, C. Liu, W. Liu and H. Lin, *Bioresource Technology*, 2022, **347**, 126362.

4. S. Nundy, A. Ghosh, R. Nath, A. Paul, A. A. Tahir and T. K. Mallick, *Journal of Hazardous Materials*, 2021, **420**, 126554.

5. H. Chen, Y. Gao, A. El-Naggar, N. K. Niazi, C. Sun, S. M. Shaheen, D. Hou, X. Yang, Z. Tang, Z. Liu, H. Hou, W. Chen, J. Rinklebe, M. Pohořelý and H. Wang, *Journal of Hazardous Materials*, 2022, **425**, 127971.
